# Study on the Effect of Hydrothermal Carbonization Parameters on Fuel Properties of Sewage Sludge Hydrochar

**DOI:** 10.3390/ma16216903

**Published:** 2023-10-27

**Authors:** Małgorzata Hejna, Kacper Świechowski, Andrzej Białowiec

**Affiliations:** Department of Applied Bioeconomy, Wrocław University of Environmental and Life Sciences, 51-630 Wrocław, Poland; malgorzata.hejna@upwr.edu.pl (M.H.); kacper.swiechowski@upwr.edu.pl (K.Ś.)

**Keywords:** organic waste, waste to energy, solid fuel, waste biomass, temperature, hydrothermal treatment

## Abstract

In the wake of economic and population growth, increased wastewater production poses a challenge related to sewage sludge treatment, which is problematic given its high moisture content, amount, and hazardous characteristics. This study focuses on the hydrothermal carbonization of sewage sludge to produce carbonous material–hydrochar, which may be an alternative to fossil fuels. The effect of process parameters, namely, temperature (180, 240, 300 °C) and duration time (30, 90, 180 min), on hydrochar properties (proximate and ultimate analysis, heating values) and process performance were studied. Obtained results indicate and confirm that hydrothermal carbonization, especially temperature increase, improves the fuel properties of carbonized sewage sludge. The highest low heating value was obtained for hydrochar derived at 300 °C in 180 min (~23 MJ × kg^−1^). The highest energy gain was noted for hydrochar derived at 240 °C in 180 min (~23%). As well as relatively high mass and energy yield in comparison to other hydrochars, these parameters are considered the most favorable for sewage sludge hydrothermal carbonization. However, high energy consumption (over 1300 kJ × g^−1^) suggests that more research on the process’s economical efficacy is required.

## 1. Introduction

In the wake of the constantly increasing population, developing economy, and consumption, the need for proper sewage treatment is growing. The solid product of the wastewater treatment process is sewage sludge (*SS*). The main producers of *SS* are North America, East Asia, and Europe, and in 2017, the world production of *SS* was estimated at 45 million Mg of dry matter [1,2]. This large quantity of *SS* poses a challenge regarding its disposal. Another problem is the hazardous characteristics of *SS* which create many environmental and health threats. For instance, it contains many pathogenic microorganisms (bacteria, viruses, and parasites), such as *V. cholera*, *Salmonella* spp., *E. coli*, and *Klebsiella* spp., which may lead to diseases spreading [3,4]. The high heavy metals content may endanger the soils and water bodies when not disposed of properly. The increasing problem with *SS* management is also related to the presence of persistent organic pollutants (POPs), including nonylphenols, polyaromatic hydrocarbons (PAHs), pharmaceuticals, and antibiotics, which do not decompose during biological aerobic and anaerobic treatment [5,6,7,8], and may be discharged to the environment when spread at a field. Another problem is created by the high moisture content reaching 80% [9], which influences transportation and treatment processes, particularly in the case of thermochemical treatment.

Growing demand for clean energy and sustainable solutions create new opportunities for *SS* management. As the 17 Sustainable Development Goals (SDGs) are to be achieved by the end of 2030, with goal no. 7 referring to affordable and clean energy, and goal no. 13 referring to climate action, sustainable solutions for *SS* management are demanded [10]. One of the issues is global greenhouse gas (GHG) emissions, with fossil fuels energy plants being the biggest contributor according to International Energy Agency (IEA) [11]. Producing solid fuel from *SS* via thermochemical processes is a way to reduce the quantity of hazardous material, increase recycling levels, reduce concern with pathogenic microorganisms and POPs, and use renewable energy [12].

One of the main goals of *SS* treatment is its hygienization and toxic compounds removal. Over the years, many methods of *SS* reuse have been applied; therefore, *SS* is not considered a waste product anymore [13]. The main methods for *SS* disposal in the EU are landfilling, incineration, agricultural use, composting, and dumping in the sea, with incineration being the most popular, for it allows effective reduction of the amount of *SS* [14,15]. One of the promising methods of *SS* treatment is thermochemical processing by torrefaction, pyrolysis, gasification, and hydrothermal carbonization (HTC), generating carbon-rich material, which can make an alternative to conventional solid fuel [16,17].

HTC is a process designed to convert biomass characterized by high moisture content; therefore, there is no need for the material to be predried [18]. HTC is very similar to the natural process of coal shaping, generating carbon-rich material called hydrochar (*HC*) in a much shorter time [19,20]. During the process, reactions such as hydrolysis, dehydrogenation, decarbonylation, decarboxylation, and polymerization occur, leading to the degradation of introduced biomass [21,22]. The main parameters influencing the HTC process and the final properties of the products are temperature, time, and pressure, with temperature being the most crucial factor. The process is usually carried out in the temperature range of 180–300 °C [23,24]. The increase in temperature provides more energy which is then used to break the bonds between the biomass molecules; hence, the efficiency of biomass conversion is being improved. At the same time, the increase in temperature results in a decrease in the yield of the solid product [19,25]. Longer residence time usually leads to increased intensity of the reactions, and the process duration ranges from a few minutes to several hours [19,24]. Pressure inside the reactor is autogenous and dependent on the temperature and initial content of water, reaching 20–100 bars [26].

*HC* derived via HTC can support the circular economy and be used as a soil amendment, adsorbent, base for carbon materials, and an energy source [27,28,29,30]. Considering the fuel properties, *HC* tends to have higher calorific value, energy yield, fixed carbon content, and lower content of volatile solids, *H*/*C*, and *O*/*C* ratios than raw feedstock, as proved by other researchers [31,32]. In the case of *SS*, slightly different correlations have been observed. For instance, according to Lu et al. [33], the carbon content decreased, as well as fixed carbon, fuel ratio, and high heating value. The same was noted by Zheng et al. [34]. As the temperature of the process rises, *HC* yield tends to decrease intensely, while the high heating value does not show significant change [35]. This may be caused by the different nature of the organic matter of the *SS* originating from the cells of activated sludge microorganisms compared to in the case of lignocellulosic biomass. Because the properties of *SS* differ significantly depending on the methods of wastewater treatment, more investigation on the HTC of *SS* is needed for a better understanding of the nature, mechanism, and process optimization.

Thermal conversion of organic materials still requires further study. It is important to better understand the possibilities for HTC use in terms of recycling, reusing, and valorizing *SS* to solid fuel. As the process temperature is the most important factor for the process’ reactions, more research on the influence of the temperature on *HC*’s properties is required. Though there are already works that have studied *HC* fuel properties of various substrates, there is still a gap in the knowledge of *HC* production performance and energy efficiency of the HTC process of anaerobically stabilized *SS*. For that reason, this study aimed to analyze the temperature and time influence on fuel properties of HCs derived from anaerobically stabilized *SS*, *HC* production process performance, and process energy consumption at the laboratory scale, providing insight into the conversion of anaerobically stabilized *SS* to solid fuel. 

## 2. Materials and Methods

### 2.1. Material (Feedstock)

Anaerobically stabilized *SS* was obtained from the wastewater treatment plant BEST-EKO Sp. z o.o. located in Rybnik, Poland. The material was collected directly from the plant and then placed in two plastic buckets with a total capacity of 20 L. To ensure homogeneity, *SS* was stirred with the use of a drill (Bosch, model Professional GSB 16 RE, Gerlingen, Germany) with a mortar stirrer. It was then divided into samples of 250 g each. The samples were placed in the freezer until further use (Electrolux, model EC5231A0W, Stockholm, Sweden) at a temperature of −27 °C.

### 2.2. Methods

#### 2.2.1. HTC Process—Hydrochar Production

**Figure 1:**
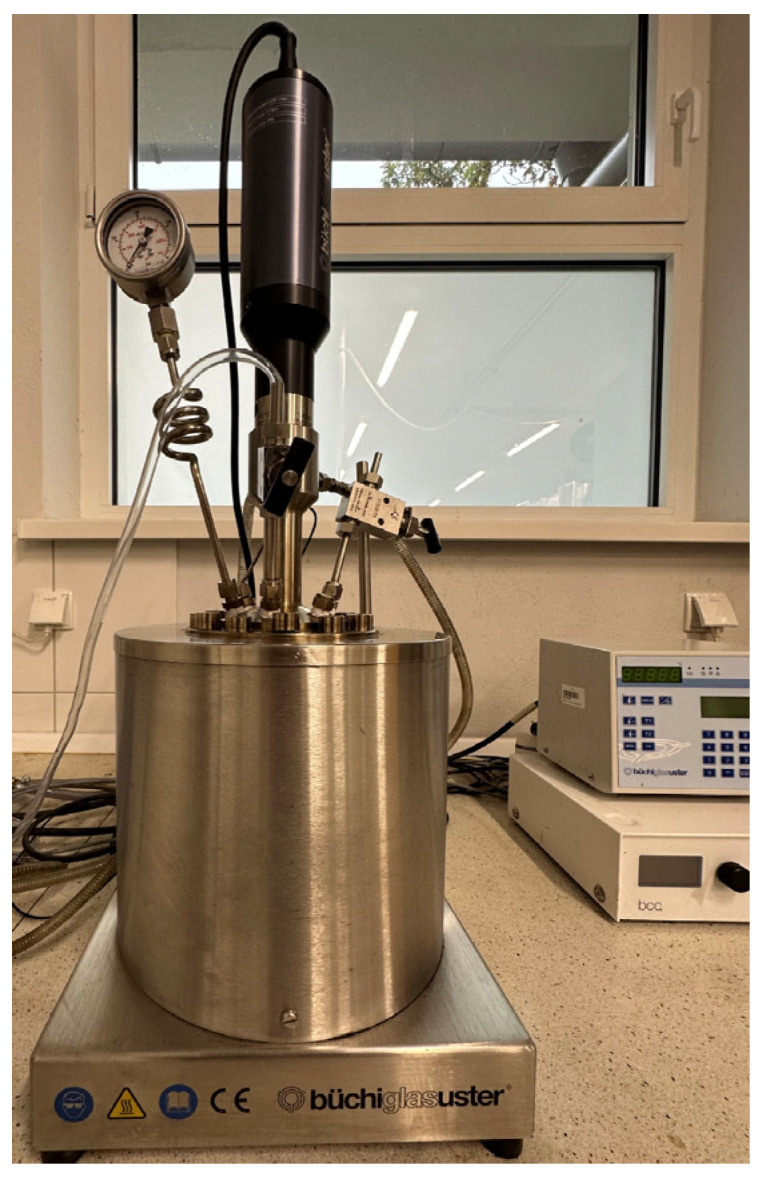
The high-temperature, high-pressure reactor used for the HTC process.

The HTC process was performed following Hejna et al. [36], using a high-temperature, high-pressure reactor (HPHT) (Büchi AG, Uster, Switzerland), presented in Figure 1. 

The material was thawed and the sample of raw *SS*, weighing 220 g, was placed into the feedstock vessel. The feedstock vessel was then placed inside the heating jacket, and the whole apparatus was closed and sealed. The stirrer speed was set to 120 rpm, and the specified temperature of the feedstock vessel was set. The temperatures at which the HTC processes were carried out were 180, 240, and 300 °C. When the temperatures of 175, 235, and 295 °C, respectively, were reached, the processes were carried out for 30, 90, and 180 min. The difference between the set temperature and the actual start temperature was caused by the extended time in which the PID temperature controller heats up by the last 5 °C. Each process was carried out three times. During the process, the pressure was generated autogenously and was measured by the manometer included in the reactor. The energy consumption was measured using an energy meter (Starmeter Instruments Co. Ltd., SK410, Shenzhen, China). After the specified process time, the reactor temperature was set to 0 °C to cool down the reactor. When the temperature of 45 °C inside the feedstock vessel was reached, the reactor was turned off, and the pressure was released. The reactor was then opened, and the processed sample was removed using a plastic spoon. The obtained sample was weighed using a laboratory scale (Radwag, MA 50.R, Morawica, Poland). The solid fraction was separated from the liquid fraction using a mesh sieve (0.063 mm), and the liquid fraction was then placed into plastic containers and frozen at a temperature of −27 °C. The solid fraction was weighed and dried using a laboratory dryer (WAMED, KBC-65W, Warsaw, Poland) at 105 °C for 24 h. Obtained *HC* samples were then ground using an electric grinder (Royal Catering, RCMZ-800, Wuppertal, Germany) and sieved using a 0.025 mm mesh sieve. Fractions below 0.025 mm were placed into plastic bags and stored for further analyses. 

#### 2.2.2. Fuel Properties Analyses

All samples were analyzed three times to ensure repeatability. Moisture content (*MC*) of raw *SS* and *HC* was determined using a laboratory dryer (WAMED, KBC-65W, Warsaw, Poland), following Świechowski et al. [37]. Thermogravimetric analysis was used to identify the volatile matter (*VM*) content, using a tubular furnace (Czylok, RST 40 × 200/100, Jastrzębie-Zdrój, Poland) [38]. The ash content (*AC*) was measured according to the procedure followed by Świechowski et al. [37], using a muffle furnace (Snol 8.1/1100, Utena, Lithuania). Fixed carbon (*FC*) was calculated as a difference between *VM* and *AC*. High heating value (*HHV*) was measured following the PN-EN ISO 18125:2017-07 standard [39], using a calorimeter (IKA, C200, Staufen, Germany). Fuel ratio (*FR*) was calculated based on Equation (1) [40].

(1) $$FR=\frac{FC}{VM}$$

where,

*FR*—fuel ratio;
*FC*—fixed carbon, %;
*VM*—volatile solids, %.

Low heating value (*LHV*) was calculated based on Equation (2) presented in Tańczuk et al. [41].

(2) $$LHV=HHV-2441.8\times (9\times \frac{H}{100})$$

where,

*LHV*—low heating value (dry basis), kJ × kg^−1^;
*HHV*—the high heating value of the analyzed material, kJ × kg^−1^;
2441.8—latent heat of water evaporation formed due to hydrogen presence in a dry material, kJ × kg^−1^;
9—hydrogen to water conversion factor;
*H*—hydrogen content in a dry material, %;
100—conversion factor.

Elemental composition was measured using an elemental analyzer (Perkin Elmer, 2400 Series, Waltham, MA, USA), following the PN-EN ISO 16948:2015-07 standard [42]. The oxygen content (O) was calculated based on Equation (3) (as a dry base) [37].

*O* = 100% − *C* − *H* − *N* − *S* − *AC* (3)

where,

*O*—oxygen content, %;
*C*—carbon content, %;
*H*—hydrogen content, %;
*N*—nitrogen content, %;
*S*—sulfur content, %;
*AC*—ash content, %.

The *H/C* and *O/C* ratios were calculated based on Equations (4) and (5), respectively [37].

(4) $$H/C=\frac{\frac{H}{1}}{\frac{C}{12}}$$

(5) $$O/C=\frac{\frac{O}{16}}{\frac{C}{12}}$$

where,

*H/C*—molar ratio of *H* to *C*;
*O/C*—molar ratio of *O* to *C*;
1—molar mass of *H*, u;
12—molar mass of *C*, u;
16—molar mass of *O*, u.

#### 2.2.3. Process Performance

Mass yield (*MY*), energy densification ratio (*EDr*), energy yield (*EY*), and energy gain (*EG*) were determined based on Equations (6)–(9), respectively [37,43].

(6) $$MY=\frac{m_{h}}{m_{r}}\;\times \;100$$

(7) $$EDr=\frac{{HHV}_{h}}{{HHV}_{r}}\;\times \;100$$

(8) EY=MY × EDr

(9) $$EG=\frac{{(HHV}_{h}-{HHV}_{r})/{HHV}_{r}}{{{(m}_{r}-m}_{h})/m_{r}}\;\times \;100$$

where,

*MY*—mass yield, %;
*m_h_*—mass of dry hydrochar after HTC process, g;
*m_r_*—mass of dry raw material before HTC process, g;
*ED_r_*— energy densification ratio, %;
*HHV_h_*—high heating value of hydrochar after HTC process, J × g^−1^;
*HHV*_r_—high heating value of raw material before HTC process, J × g^−1^;
*EY*—energy yield, %;
*EG*—energy gain, %.

#### 2.2.4. Statistical Analysis

To find statistically significant differences, the two-way analysis of variance (ANOVA) with post hoc Tukey test was performed at the level of α = 0.05, using Statistica 13.0 software (TIBCO Software Inc., Palo Alto, CA, USA). Raw results obtained during statistical analysis are presented in Appendix A (Table A1, Table A2, Table A3, Table A4, Table A5, Table A6, Table A7, Table A8, Table A9, Table A10, Table A11, Table A12, Table A13, Table A14, Table A15, Table A16 and Table A17).

## 3. Results and Discussion

### 3.1. Properties of Raw Sewage Sludge

The *SS* used for this research contained 82.10 ± 0.65% of moisture, which is favorable, for it is claimed that the ideal *MC* for the HTC process is 75–90% [44]. A similar *MC* in *SS* was noted by Pulka et al. [45], with a result of 79.70%. Table 1 contains the results of proximate and ultimate analysis, and heating values of raw *SS*. Received results were compared with the results obtained by other researchers [46,47,48]. *VM* content in *SS* appeared to be higher than in *SS1*–*SS3*, while both *AC* and *FC* values were lower. As for the ultimate analysis, the *C*, *H*, and *S* contents were higher compared to the given literature sources. The *N* and *O* contents reached intermediate values of 3.80% and 14.58%, respectively. The *HHV* of *SS* reached almost 21 MJ × kg^−1^ and was higher than the results of other researchers (Table 1), due to relatively high *C* content. *LHV* calculated for both dry basis and as received was the highest for *SS* investigated in this study (Table 1). A detailed discussion of the influence of particular parameters on fuel properties is presented further in the paper.

### 3.2. Performance of the HTC Process

The *SS* was processed in the reactor with a total volume of 600 mL. The material inside the reactor was heated to specific temperatures with the same power, and the heating rates varied from 3.98 to 5.46 °C × min^−1^. The heating rate differed depending on the setpoint temperature, and obtained differences might have been the result of the used PID controller characteristics and possible occurrence of endo/exothermal reactions during material decomposition. After the end of the process, the reactor was cooled down to temperatures of around 45 °C, with heating rates varying from 1.82 to 3.24 °C × min^−1^ (Table 2). 

For each process, 220 g of wet *SS* was used. Due to the moisture content of *SS* being 82.2%, the mass of total solids in the reactor was 39.16 g. As a result of the HTC process, the process slurry and process gas were obtained. The slurry consisted of solids particles (*HC*) and liquids (process water). In Table 2, the mass yields (*MY*) of each product are summarized. The mass yields refer to the initial mass of wet *SS* used for the HTC. Results show that with increasing temperature and process time, solids *MY* decreased from 11.45% to 3.97% in favor of liquid fraction, for which an increase in *MY* was observed from 64.73% to 73.57% for 180 °C, 30 min, and 300 °C, 180 min, respectively (Table 2). The change in *MY* of produced gas did not show any specific trend; therefore, it can be assumed that an increase in temperature and process time results in the conversion of solid matter to liquid more than to gas. Also, the pressure of the process increased with higher temperatures, reaching the average of 89 bars for 300 °C, 180 min (Table 2). The obtained pressure values correspond well with the information given by Nizamuddin et al. [49], according to whom pressure during the HTC ranges from 20 to 100 bars.

**Figure 2:**
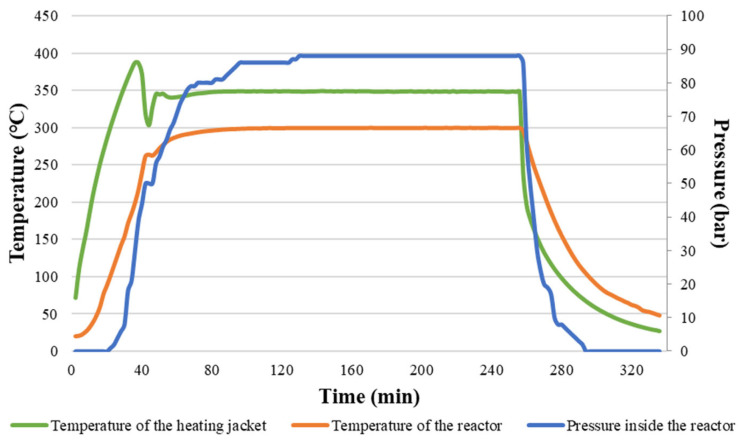
An example of the average temperature and pressure patterns during the process at 300 °C in 180 min.

Figure 2 presents an example of the process development regarding changes in the temperatures of the heating jacket inside the reactor and the pressure. 

The average heating rate was 4.85, 5.23, and 3.89 °C × min^−1^ for 180, 240, and 300 °C, respectively. The cooling rate increased with both temperature and time, reaching the average of 2.04, 2.83, and 3.14 °C × min^−1^ for 180, 240, and 300 °C, respectively.

**Figure A1:**
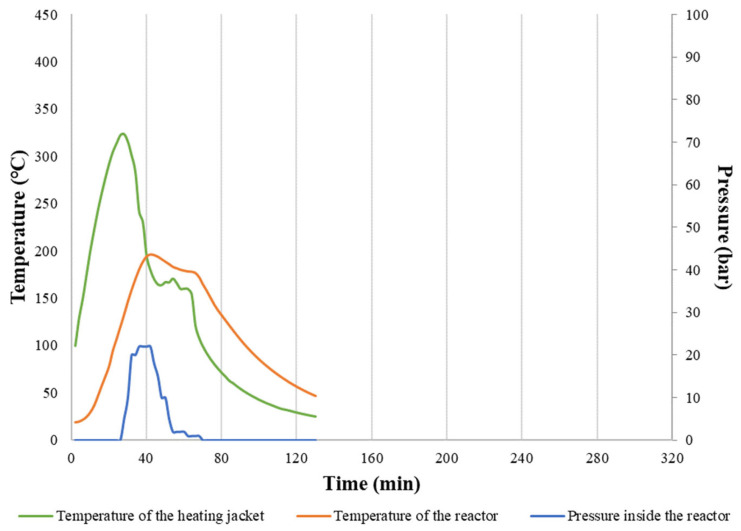
Temperature and pressure patterns during the process at 180 °C in 30 min.

**Figure A2:**
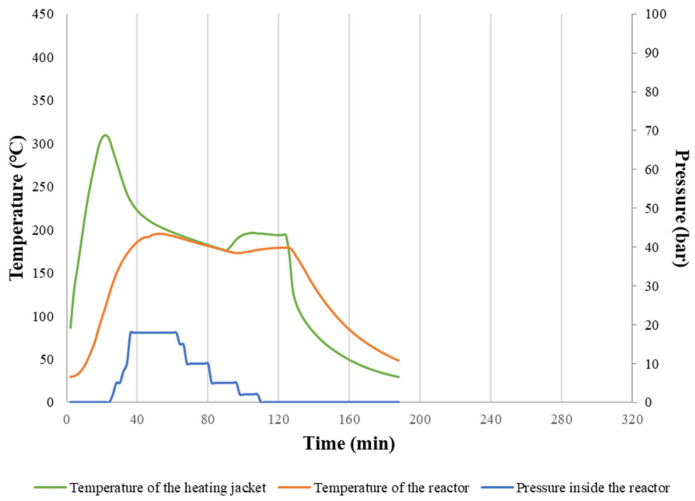
Temperature and pressure patterns during the process at 180 °C in 90 min.

**Figure A3:**
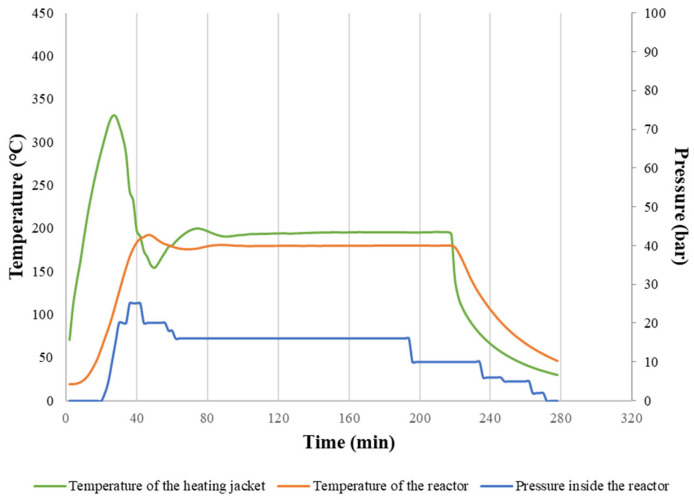
Temperature and pressure patterns during the process at 180 °C in 180 min.

**Figure A4:**
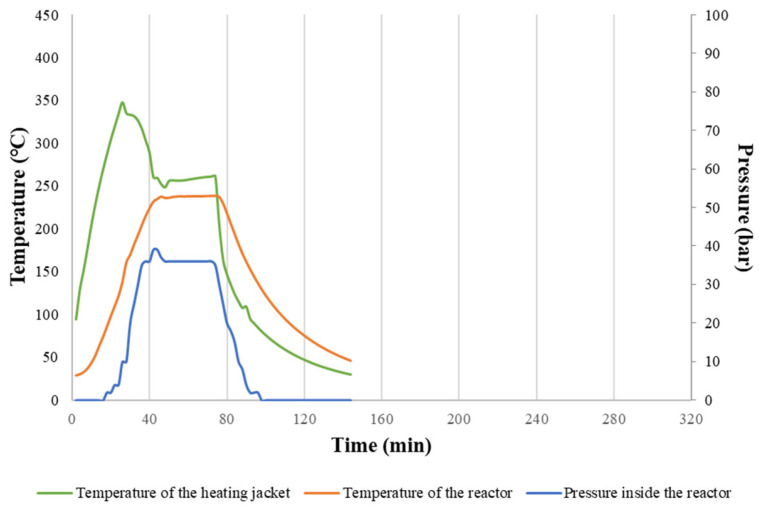
Temperature and pressure patterns during the process at 240 °C in 30 min.

**Figure A5:**
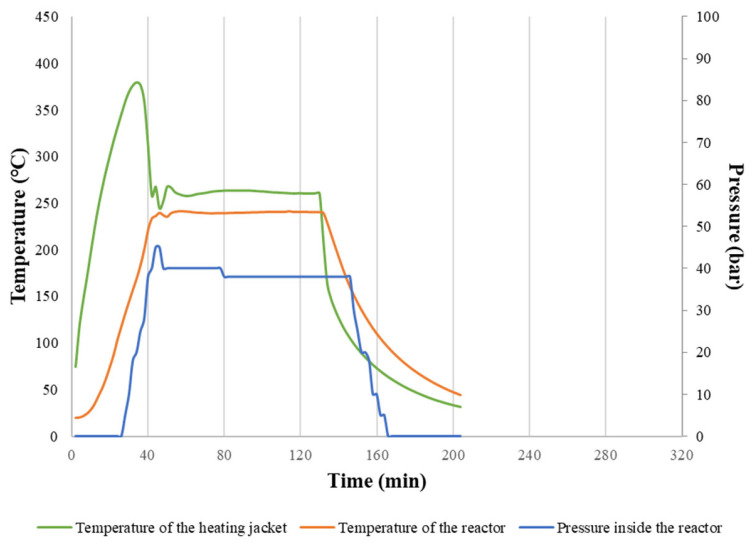
Temperature and pressure patterns during the process at 240 °C in 90 min.

**Figure A6:**
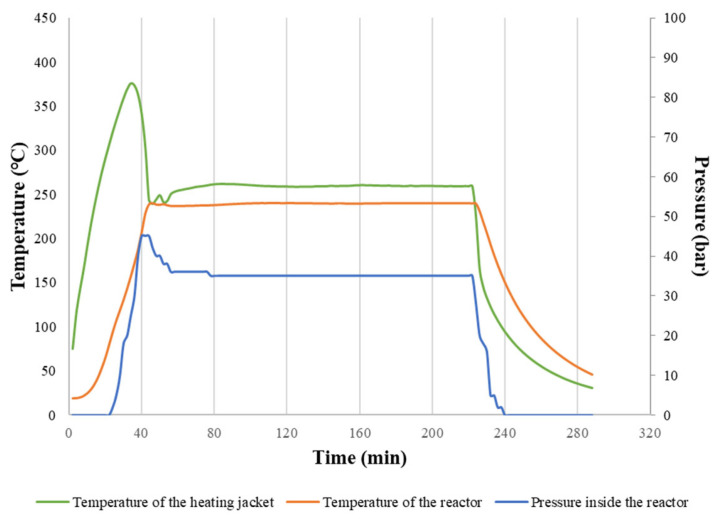
Temperature and pressure patterns during the process at 240 °C in 180 min.

**Figure A7:**
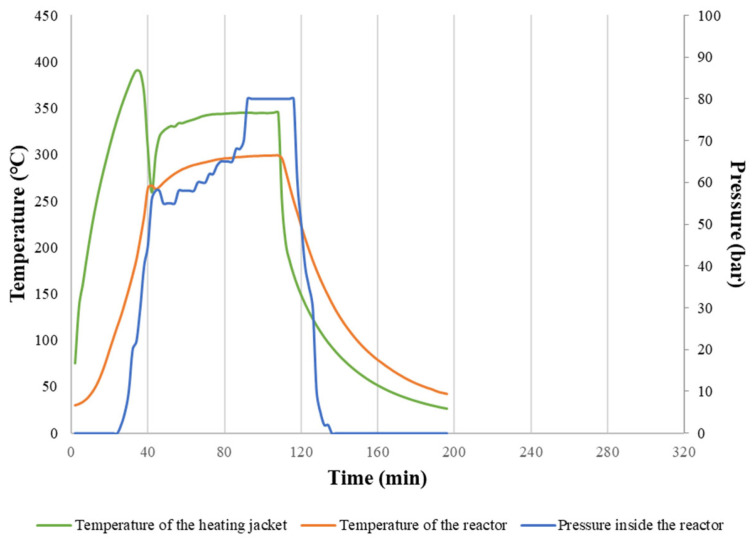
Temperature and pressure patterns during the process at 300 °C in 30 min.

**Figure A8:**
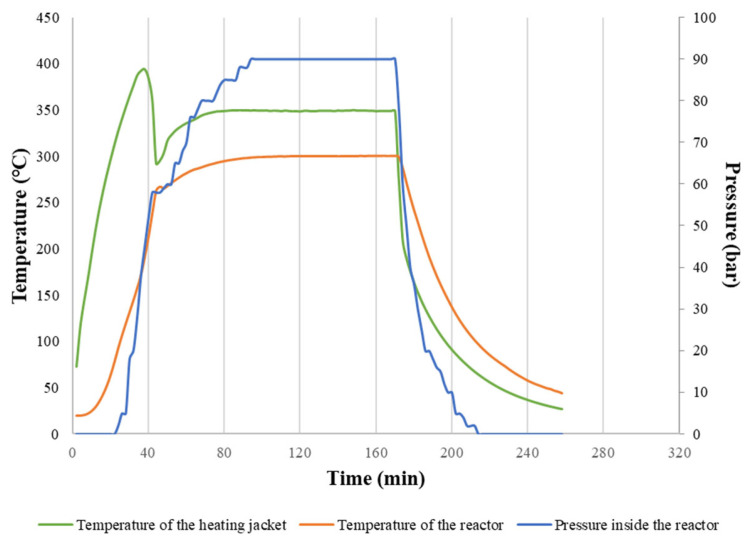
Temperature and pressure patterns during the process at 300 °C in 90 min.

Graphs visualizing the average patterns of temperature and pressure during the process of HTC for different parameters are summarized in Appendix B (Figure A1, Figure A2, Figure A3, Figure A4, Figure A5, Figure A6, Figure A7 and Figure A8). According to the graphs, pressure depended on the set temperature, which confirms the reports given in the literature [50]. The increase in pressure was related to temperature, headspace in the reactor, and the amount of gas being produced during HTC, hence the process of material decomposition.

### 3.3. Fuel Properties of Hydrochars

Generally, the temperature of the HTC process has a more considerable influence on *HC*’s fuel properties and pressure generated during the process than retention time [36]. Conducted research showed that in the case of *SS*, the effect was not significant in the case of time variations (Table 3) (Table A1, Table A2, Table A3 and Table A4).

The *VM* content decreased significantly (*p* < 0.05) from both raw *SS* and *HC* derived at 180 °C in 30 min to *HC* derived at 300 °C in 180 min, reaching the lowest value of 56% (Table 3, Table A1). The lower the *VM* content, the higher quality of the fuel, because *VM* leads to tar production; hence, problems within combustion systems occur [51]. A decrease in *VM* content was also observed by other researchers. For example, *VM* content of *HC*s derived from waste straw decreased with both temperature and residence time [52], at the same time being higher than results obtained from *SS* in this study. Results obtained by Sobek et al. [52] were also lower than *VM* content in *HC* obtained from *SS* in 30 min 180 and 240 °C, reaching 70.50 and 63.25%, respectively. When compared to coal, which contains up to 44% of *VM*, the results are still not satisfying [53,54]. 

The *FC* content decreased in comparison with raw *SS* and was the highest (4.20%) for *HC* derived at 300 °C in 180 min, and the upward trend can be observed as well (Table 3). The increase in the *FC* content is due to the temperature increase, which causes the devolatilization process of *VM*, hence the increase in the amount of remaining solid carbon [55]. Received results are very low when compared with lignite coarse coke (69.90% of *FC*), and high levels of *FC* are in favor [56,57]. Furthermore, in the research conducted by Lee et al. [53], the *FC* in *HC*s derived from *SS* was 11.43% and 13.52% for temperatures of 180 °C and 240 °C, respectively. *SS* contains a lot of cellulose, hemicellulose, and lignin (87% on average) due to the high quantity of toilet paper, which is the paramount organic component of municipal sewage, and it is assumed that toilet paper contains approximately 85% cellulose [58]. Research conducted by Demirbaş [59] suggests that the hemicellulose, cellulose, and lignin ratio influence the FC content, with cellulose being the component that decreases the *FC* parameter. Therefore, it may be concluded that *SS* used for this research contains even more cellulose than the approximate values.

*FR* of all obtained *HC*s was <2.5 (Table 3), which indicates that the material can be satisfactorily used in pulverized fuel-burning systems [60]. It can be also observed that the *FR* increased with temperature rise. No statistically significant differences were observed for time changes (*p* > 0.05) (Table A3).

The presence of alkali metals in ash may cause damage in combustion installations [60]. Furthermore, the higher the *AC*, the more waste is generated during combustion. Therefore, the *AC* is an important indicator of fuel quality. The highest *AC* was noted for the *HC* derived at 300 °C in 180 min (~40%) (Table 3), which implies that the process of HTC and temperature increase result in higher *AC* values. Even higher results for hydrothermally treated *SS* were obtained by Wilk et al. [61], with *AC* of 52.04% for *HC* derived at 200 °C in 120 min. The change in *AC* is the result of two reasons, namely, extracting inorganics from the matrix of biomass into the water, hence removing it from the solid fraction, and organic matter breaking down into the liquid phase, resulting in decreased *MY*, hence densifying the ash in the *HC* [62]. For comparison, *HC* derived from dead leaves at a temperature of 240 °C in 30 min contained 19.19% of ash, and *HC* derived from watermelon peel at 260 °C in 60 min contained 5.33% of ash [63,64]. This proves the statement posed by Syed-Hassan et al. [16] that *SS,* on average, is characterized by a much higher *AC* than coal and other types of biomasses.

The *C* content increased after hydrothermal treatment, as well as due to the increased temperature of the process, reaching the maximum of almost 49% for *HC* derived at 300 °C in 90 min with an increase of 5.20 percent points concerning raw *SS* (Table 1, Table 4). The increase in *C* content is caused by decaying carbon bonds on the surface of biomass and volatilization or degradation of *H* and *O*-reach compounds [53].

No significant differences were observed for *H* content values (*p* > 0.05) (Table A6); values decreased with temperature and duration time increase. The lowest *H* value was noted for *HC* derived at 300 °C in 30 min (Table 4). Noticeably, the *O* content intensely decreased, reaching the minimum of 0.51% for *HC* derived at 300 °C in 90 min (Table 4). Similar tendencies for *C*, *H*, and *O* contents are commonly observed for *HC* derived from different materials, such as chicken manure, dairy manure, swine manure, and food waste [36,60,65]. The statistically significant effects of temperature and time on the change in elemental composition are given in Appendix A in Table A5, Table A6, Table A7, Table A8 and Table A9.

**Figure 3:**
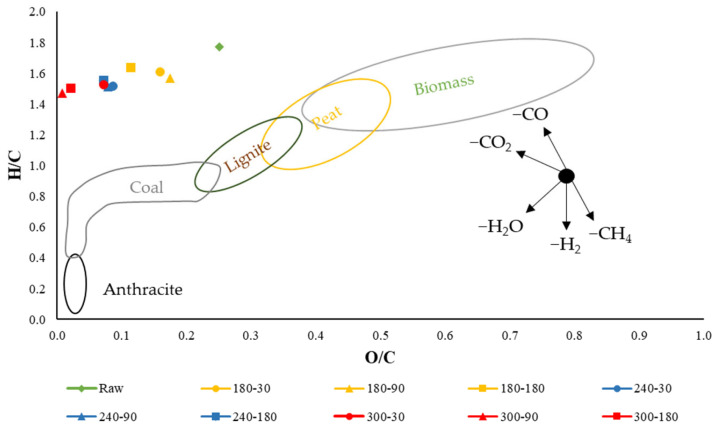
Van Krevelen diagram for hydrochars and raw sewage sludge.

During the thermochemical process of biomass decomposition, many reactions take place. Among others, general reactions like decarbonization, dehydrogenation, and deoxygenation result in mass loss of *C*, *H*, and *O* [66]. Therefore, though the elemental composition of *HC*s changes, the content of particular elements is relative, and the relative increase or decrease in specific elements’ percentage share depends on processed material and courses of particular reactions taking place during HTC. The fact that some reactions took place during the HTC process can be found using the van Krevelen diagram (Figure 3). 

The diagram was introduced in 1950 by D.W. van Krevelen to help understand coal thermochemical processes, where reactions like dehydration, dehydrogenation, decarboxylation [67], decarbonylation, demethylation, and demethanation take place [68]. Those reactions result in the production of liquid and gas products (respectively, H_2_O, H_2_, CO_2_, CO, CH_3_, and CH_4_) shifting molecular ratios in specific directions [68]. It is well known that HTC leads to a decrease in *H*/*C* and *O*/*C* ratios, which is mainly due to reactions of dehydration and decarboxylation [69]. Though separate reaction mechanisms are well known, the reaction network during HTC is not fully understood. The relative significance and the course of particular reactions primarily depend on the type of processed material and process conditions [70]. In Figure 3, the molecular ratios for obtained *HC*s are presented with a visualization of ratios expected for common solid biofuels [71] and general vectors of chemical reactions [68]. In general, the lower the ratios of *H*/*C* and *O*/*C*, the better the fuel quality of the material [69]. 

For studied *SS*, the molar ratios of *H*/*C* and *O*/*C* were 1.77 and 0.25, while for obtained *HC*s, these values varied from 1.61 to 1.50 and from 0.16 to 0.01. As a result, they did not fit into any common solid biofuel group (Figure 2). It can be seen that with increasing temperature and retention time, both molar ratios decreased as a result of carbonization. The *H*/*C* ratios were similar to the initial value; however, the significant decrease in the *O*/*C* ratio may indicate the intensive deoxygenation processes mostly caused by the decarboxylation and dehydration—a loss of two atoms of oxygen in each molecule. Similarly to the result of Jellali et al. [72], the main reaction responsible for the carbonization (the increase in *C* content) with increasing temperature was dehydration (-H_2_O). The higher degree of *O*/*C* ratio decrease than in the case of *H/C* may indicate that the HTC process may be pretreatment before the gasification with a high yield of H_2_, and CO in the syngas, but it requires further investigation. The obtained results are partly in agreement with other studies. In the work of Mendoza Martinez et al. [73], the primary sludge was hydrothermally carbonized at temperatures of 180–240 °C for 3 h and pressure of 10–35 bar. The atomic ratios of *SS* (*H*/*C* and *O*/*C*) were 1.84 and 0.78, while for *HC*s, atomics ratio decreased to 1.62 and 0.67 (a smaller degree of the *O*/*C* ratio decrease than in the present research). Mendoza Martinez et al. [73] also found that the main reaction pathways with increasing temperature of HTC were dehydration (-H_2_O) and demethylation (-CH_3_), while the role of decarboxylation (−CO_2_) was relatively small, which is opposite in the present research. In the work of Wang et al. [69], organic sludge was hydrothermally carbonized at temperatures of 180–240 °C and retention times of 60–240 min. Though the sludge was characterized by high initial values of atomic ratios *H*/*C* and *O*/*C*, respectively, 2.08 and 0.58, a similar decreasing trend with increasing temperature and retention time of the HTC process was found. Wang et al. [69] also found that the dominant effect on the organic sludge dehydration was the temperature, while the retention time effect was negligible.

The *N* content decreased with the temperature increase, significantly (*p* < 0.05) falling from about 3.80% for raw *SS* (Table 1) to about 2.50% for *HC* derived at 300 °C in 180 min (Table 4). Those values are lower than the results obtained by Lee et al. [53], where *HC* derived from *SS* at 180 °C and 240 °C in 30 min contained 6.78% and 7.15% of *N*, respectively. However, the *N* content in *HC* obtained from sweet potato waste and fruit waste was lower [66,74].

As for the S content, there were no significant changes (*p* > 0.05) (Table A8) with temperature and time increase, and the average value was 2.76% (Table 4). Generally, the amounts of N and S in *SS* are considerably higher in comparison with other biomass materials, in which the average share of N reaches 0.94% and S 0.24% [16]. The high contents of those elements pose challenges and threats related to SOx and NOx emissions [51,55]. According to Yao et al. [75], the S content in solid fuel obtained during HTC is one of the limiting factors for its energy use. Additionally, it has been observed that the S content in soils tends to decrease [76]. While this is an essential component, its deficiencies result in, among others, delayed plant growth, discoloration, and smaller leaves [77]. Furthermore, S availability improves N uptake by plants. High amounts of S and N in obtained *HC* may suggest further use in soil fertilization. This could also refer to the liquid fraction obtained during the HTC process, as it was proved that some valuable nutrients, such as phosphorus and nitrogen, are solubilized into the liquid phase, which creates a possibility for the recovery of these compounds [78].

**Figure 4:**
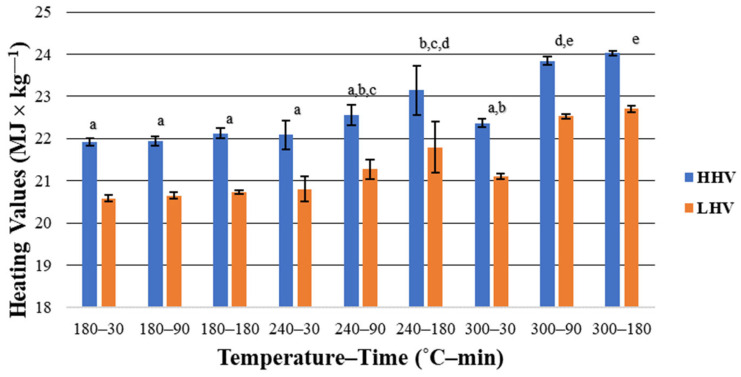
High and low heating values of hydrochars—average value ± standard deviation. Letters (a–d) indicate of statistically significant differences.

*HHV* is described as the highest possible energy being released through the process of one fuel unit’s full oxidation [79]. As shown in Figure 4, an increase in both temperature and HTC duration time had a positive influence on the HHV. 

The lowest *HHV* was noted for *HC* derived at the lowest HTC parameters (180 °C, 30 min), reaching less than 22 MJ × kg^−1^, whereas *HC* obtained at the highest parameters of 300 °C and 180 min was characterized by the highest result (~24.02 MJ × kg^−1^). The difference between the highest *HHV* result and the *HHV* of raw *SS* was 3.07 MJ × kg^−1^. A similar phenomenon was observed by Volpe et al. [50], who treated *SS* with HTC at 190 °C and 210 °C in 60 min and 180 min. *HHV* for *SS* and all treated samples remained stable, reaching ~16.50 MJ × kg^−1^. The reason for such behavior may be the increase in the content of inorganic material. The *HHV* value of obtained *HC*s may be considered high. For comparison, *HHV* of *HC* derived from yard waste at temperatures of 160–200 °C in a retention time of 120–1440 min was 15.72–24.59 MJ × kg^−1^ [80].

*LHV* is useful when determining the real energy potential of biomass and refers to the amount of heat being released during complete combustion, including vaporization heat of the water that remains in the product [55,81]. The same tendency for the *HHV* was observed for the *LHV* (Figure 4). The highest *LHV* (dry basis) was noted for *HC* derived at 300 °C in 180 min, with the result of 22.71 ± 0.07 MJ × kg^−1^. For comparison, the average *LHV* of food/yard waste and textiles is 14.60 MJ × kg^−1^ and 19.09 MJ × kg^−1^, respectively [81]. *LHV*, as received, ranged from 0.82 MJ × kg^−1^ for *HC* derived at 300 °C in 180 min to 2.69 MJ × kg^−1^ for *HC* derived at 180 °C in 30 min. Differences between particular values were significant, especially for higher temperatures (*p* < 0.05) (Figure 4, Table A11).

### 3.4. Hydrochar and HTC Energy Yields

**Figure 5:**
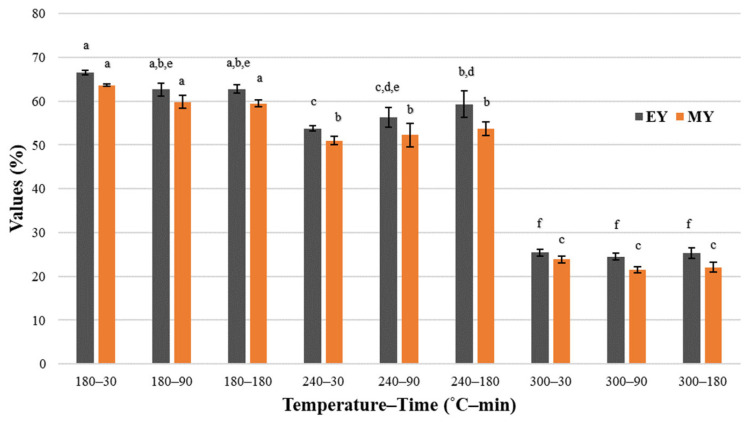
Mass and energy yield in temperature and HTC duration time—average value ± standard deviation. The lowercase letters (a–f) indicate statistically significant differences between groups; groups marked with the same letter do not differ.

Higher pressure of the processes led to lower *HC* yield, which can be seen in Figure 5.

*MY* was the highest (~63%) for *HC*s derived at the lowest temperature (180 °C), and the lowest (less than 21%) for the highest temperature (300 °C). The described decrease in *MY* may be caused by the process of decarboxylation, which is in accordance with the significant *O*/*C* ratio decrease, forming organic matter, which is soluble in water. Decomposition and depolymerization of cellulose and hemicellulose present in *SS* lead to enhanced generation of the liquid and gaseous products of HTC [58,82].

The same tendency and very similar results were obtained in the case of the *EY*, which describes the amount of energy remaining in *HC*. A decrease in the *EY* occurs due to the decomposition and conversion of material into liquid and gas products [83]. The boundary values were ~67% and ~25%. This dependence has also been observed in the literature. For instance, Lee et al. (2019) [53] noted that a temperature increase from 180 °C to 270 °C led to a decrease in *MY* from 93.13% to 40.78%. The same phenomenon was observed for *HC* derived from beet pulp [61]. However, in both cases, *MY* was higher compared to results obtained during this research, and the decrease was not so considerable. Li et al. (2022) [65] observed a decrease in both *MY* and *EY* for *HC* obtained from animal manures, with the lowest values reaching ~20%, hence similar to those obtained in this study. These results suggest that, in terms of *MY* and *EY*, HTC of *SS* is not the most optimal route for its conversion, and that the temperature of the process should be lower to prevent the release of organic matter into a liquid fraction.

*EDr* describes the changes in the energy content of *HC* concerning the raw material. As shown in Table 5, *EDr* increased with both time and temperature. 

The lowest value was obtained for HC derived at 180 °C in 30 min (~104%), and the highest value for *HC* was derived at 300 °C in 180 min (~114%). This tendency was also observed for *HC* derived at 180, 200, and 220 °C in 60, 120, 180, and 240 min from beet pulp. The highest *EDr* was seen for the highest process parameters and reached 147% [61]. Those values suggest that the results of *EDr* obtained in this study are relatively low.

In general, *EG* tends to increase with increasing time and temperature of the process [43]. However, here, the highest *EG* was obtained for *HC* derived at 240 °C in 180 min (almost 22%), and the lowest for *HC* derived at 300 °C in 30 min (~8%) (Table 5). *HC* derived from eucalyptus tree residues at temperatures 250–300 °C in time of 20–60 min had *EG* in the range of 51–63%. Previously conducted research on chicken manure in the same process parameters showed that *EG* can reach up to 97.60% [36]. Therefore, it may be concluded that the results obtained in this research are not satisfactory in the case of both *EDr* and *EG*.

**Figure 6:**
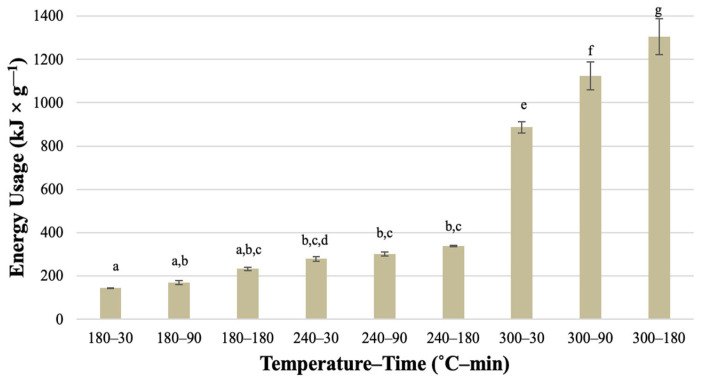
Energy usage of the HTC process to the mass of dry hydrochar obtained after the process—average value ± standard deviation. The lowercase letters (a–g) indicate statistically significant differences between groups; groups marked with the same letter do not differ.

As presented in Figure 6, the energy usage (*Eu*) increased with both temperature and time, reaching over 1300 kJ × g^−1^. 

**Figure 7:**
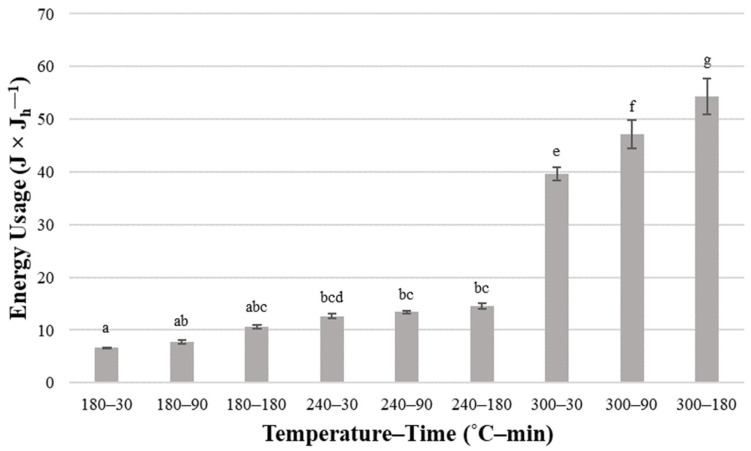
Energy usage of the HTC process relative to the unit of energy available in the unit of dry hydrochar obtained after the process—average value ± standard deviation. The lowercase letters (a–g) indicate statistically significant differences between groups; groups marked with the same letter do not differ.

However, the most considerable increase was observed for processes conducted at 300 °C. The highest result was over nine times higher than the lowest result obtained for the *HC* derived at 180 °C in 30 min. Energy usage of the HTC process relative to the unit of energy available in the unit of dry *HC* obtained after the process (*Eue*) was also calculated (Figure 7). The same tendency for energy usage relative to the mass of dry *HC* was observed, with the highest result of over 50 J × J_h_^−1^. 

Statistically significant differences were mainly noted for energy usage by processes conducted at 300 °C (Table A16 and Table A17). The difference between 300 °C and lower temperatures can also be seen in Figure 6 and Figure 7. This suggests that the HTC process of *SS* should be conducted at lower temperatures to provide profitability. For comparison, the chicken manure processed using the same reactor, sample size, and operating conditions (temperature, time) was characterized by much smaller energy consumption concerning the produced *HC* [36]. To produce 1 g of *HC* from chicken manure, from 75 to 425 kJ is needed for 180–30 and 300–180 variants, respectively, while to produce 1 J of energy in *HC* requires from 5 to 18 J [36]. This shows that feedstock properties are an important factor affecting the energy efficiency of the HTC process. 

However, it has to be noted that this research, as well as [36], was conducted at a laboratory scale; thus, the heat loss was high, especially for the 300 °C variant. At the industrial scale, the HTC process would be less energy-demanding due to better isolations and the presence of heat exchangers between input feedstock and output slurry. In addition, the review works of [84,85,86] showed that it can be cost-effective to combine HTC with the anaerobic digestion (AD) of *SS,* which is usually implemented in the wastewater treatment plant. The SS is converted into biogas for electricity and heat production, while digestate is future-processed in HTC to produce solid fuel using residual heat from the combined heat and power unit (CHP). Moreover, the HTC process water can be reversed to the AD process, increasing methane production [84,85,86]. Though more research is needed on the economic aspects of the combined HTC and AD, the work of Merzari et al. [84] showed that a positive energy balance of HTC–AD can be found for 180–200 °C and 15–30 min. At higher temperatures and longer times, the process water can become toxic and hard to biodegrade in AD [84]. This, and the fact that in this research the highest *MY* and *EY* with the lowest energy demands were found at 180 °C, indicates a need for further optimization research in a temperature range of 160–200 °C combined with parallel AD of HTC process waters and its effect on methane production.

## 4. Conclusions

In this paper, hydrothermal carbonization was proposed as a method of sewage sludge utilization, resulting in obtaining energy-rich carbonous material—hydrochar. The suggested solution could be an answer to the growing demand for clean energy, as well as for problems related to the management of difficult-to-manage waste, such as sewage sludge. The research showed that process temperature had the most significant impact on process performance and fuel properties of hydrochar. The best fuel properties were noted for hydrochar obtained at the highest hydrothermal carbonization parameters (300 °C, 180 min), with a high heating value reaching ~24.02 MJ × kg^−1^ and carbon content of ~48%. The research also focused on energy consumption during the process of hydrothermal carbonization, which has not been thoroughly studied before in the case of sewage sludge.

Surprisingly, the obtained hydrochar contained little fixed carbon, with a maximum value of 4.20%, and high ash content (up to ~39%). As a result, low content of fixed carbon favored high content of volatile matter, probably due to the presence of high cellulose content in processed sewage sludge. Hydrothermal carbonization improved the fuel properties of sewage sludge, but the process was characterized by high levels of energy usage, questioning the viability of this solution. However, it must be noted that the process was conducted at the laboratory scale, and a bigger scale, as well as using a heat exchanger, will significantly reduce the losses and increase the process efficiency. The highest losses were observed for processes run at 300 °C. Therefore, to use hydrochar derived from sewage sludge as a solid fuel, lower temperatures are preferable, and further research on energy balance is essential. To maximize the profitability of the proposed solution, it is suggested to combine hydrothermal carbonization of sewage sludge with anaerobic digestion of sewage sludge, where biogas produced from sewage sludge can be used as a source of energy for the hydrothermal carbonization process. Considering determined fuel properties and energy gain, with the highest value noted for hydrochar derived at 240 °C in 180 min (~23%), as well as relatively high mass and energy yield in comparison to other hydrochars, 240 °C and 180 min are considered the most favorable parameters to produce hydrochar from sewage sludge for solid fuel production. Furthermore, high nitrogen and sulfur contents were noted in the produced hydrochars, which is unfavorable due to adverse effects on the environment and combustion systems. However, this implies that hydrochar obtained from sewage sludge may be satisfactorily used as a soil fertilizer, thus opening up the next alternative for hydrochar utilization. Knowing that higher temperatures of hydrothermal carbonization improve fuel quality of produced hydrochar but also potentially may lead to the production of toxic compounds, as well as require much more energy, more research on possible hydrothermal carbonization products’ applications and optimizations are needed. Therefore, the next research works should not only focus on solid fuel properties but also on process water quality and its parallel utilization.

## Figures and Tables

**Table 1:** Results of proximate, ultimate, and heating value analysis for raw sewage sludge in this (average value ± standard deviation) and other studies.

Properties	SS (This Study)	Other Studies		
		SS1 [46]	SS2 [47]	SS3 [48]
Proximate Analysis (%) *				
*VM*	67.54 ± 0.32	60.05	38.64	51.80
*AC*	28.79 ± 0.11	28.74	57.23	43.10
*FC*	3.67 ± 0.39	11.21	4.13	5.10
Ultimate Analysis (%) *				
*C*	43.60 ± 0.05	36.14	21.28	29.02
*H*	6.44 ± 0.07	5.48	3.61	4.15
*N*	3.80 ± 0.04	6.19	3.70	4.50
*S*	2.79 ± 0.02	1.13	0.58	1.17
*O*	14.58 ± 0.12	22.20	13.60	18.09
Heating Value (MJ × kg^−1^)				
HHV *	20.95 ± 0.04	16.24	10.66	13.50
LHV *	19.53 ± 0.05	15.03	9.40	12.30
LHV **	3.49 ± 0.13	2.69	1.76	2.25

* As dry base. ** As received (*MC* = 82.10%).

**Table 2:** The average performance of each process variants’ combination.

Temperature (°C)	Time (min)	Pressure (bar)	Heating Rate (°C × min^−1^)	Cooling Rate (°C × min^−1^)	MY of Solids (%)	MY of Liquids (%)	MY of Gas (%)
180	30	21	4.82 ± 0.86	1.82 ± 0.15	11.45 ± 0.05	64.73 ± 2.13	23.82 ± 2.18
	90	18	4.91 ± 0.43	2.07 ± 0.21	10.77 ± 0.26	70.42 ± 1.99	18.81 ± 2.10
	180	21	4.82 ± 0.56	2.22 ± 0.42	10.70 ± 0.14	71.51 ± 3.00	17.79 ± 3.07
240	30	40	5.46 ± 0.62	2.77 ± 0.35	9.18 ± 0.18	70.76 ± 2.21	20.06 ± 2.37
	90	41	5.30 ± 0.35	2.83 ± 0.27	9.40 ± 0.47	69.96 ± 2.86	20.63 ± 3.33
	180	43	4.94 ± 0.27	2.89 ± 0.53	9.67 ± 0.29	69.34 ± 3.72	21.00 ± 3.79
300	30	85	3.95 ± 0.41	3.13 ± 0.47	4.29 ± 0.13	71.26 ± 1.38	24.45 ± 1.51
	90	89	3.75 ± 0.11	3.05 ± 0.71	3.88 ± 0.12	72.97 ± 1.68	23.15 ± 1.73
	180	89	3.98 ± 0.20	3.24 ± 0.13	3.97 ± 0.20	73.57 ± 1.17	22.46 ± 1.04

**Table 3:** Proximate analysis of hydrochars—average value ± standard deviation.

Temperature (°C)	Time (min)	Pressure (bar)	VM (%) *	FC (%) *	FR (−) *	AC (%) *
180	30	21	65.53 ± 0.30	2.25 ± 0.37	0.03 ± 0.01	32.23 ± 0.25
	90	18	65.23 ± 0.33	1.66 ± 0.47	0.03 ± 0.00	33.11 ± 0.52
	180	21	64.22 ± 0.45	1.64 ± 0.24	0.03 ± 0.01	34.14 ± 0.48
240	30	40	60.43 ± 0.43	2.90 ± 0.52	0.05 ± 0.01	36.67 ± 0.26
	90	41	61.18 ± 0.63	1.90 ± 0.56	0.03 ± 0.01	36.91 ± 0.21
	180	43	61.80 ± 0.32	1.88 ± 0.44	0.03 ± 0.01	36.31 ± 0.32
300	30	85	56.84 ± 0.54	3.40 ± 0.54	0.06 ± 0.01	39.76 ± 0.18
	90	89	57.36 ± 0.39	3.41 ± 0.52	0.06 ± 0.01	39.23 ± 0.43
	180	89	56.42 ± 0.51	4.20 ± 0.47	0.07 ± 0.01	39.38 ± 0.31

* As dry base.

**Table 4:** Elemental analysis of derived hydrochars—average value ± standard deviation.

Temperature (°C)	Time (min)	C (%) *	H (%) *	N (%) *	S (%) *	O (%) *
180	30	45.53 ± 0.40	6.11 ± 0.08	3.73 ± 0.21	2.74 ± 0.15	9.66 ± 0.12
	90	44.69 ± 0.51	5.85 ± 0.08	3.19 ± 0.20	2.77 ± 0.09	10.40 ± 0.42
	180	46.57 ± 1.45	6.36 ± 0.12	3.27 ± 0.10	2.63 ± 0.02	7.03 ± 0.30
240	30	46.08 ± 0.58	5.83 ± 0.22	3.24 ± 0.05	2.91 ± 0.09	5.27 ± 0.27
	90	46.53 ± 0.22	5.86 ± 0.15	2.96 ± 0.11	2.83 ± 0.15	4.90 ± 0.34
	180	47.67 ± 0.69	6.18 ± 0.11	2.70 ± 0.09	2.61 ± 0.13	4.53 ± 0.83
300	30	44.83 ± 0.06	5.70 ± 0.11	2.76 ± 0.09	2.67 ± 0.15	4.29 ± 0.28
	90	48.80 ± 1.51	5.98 ± 0.34	2.77 ± 0.21	2.70 ± 0.15	0.51 ± 0.31
	180	47.78 ± 0.57	5.98 ± 0.15	2.50 ± 0.03	2.99 ± 0.33	1.37 ± 0.71

* As dry base.

**Table 5:** Energy densification ratio and energy gain in temperature and time—average value ± standard deviation.

Temperature (°C)	Time (min)	EDr (%)	EG (%)
180	30	104.65 ± 0.33	12.79 ± 1.00
	90	104.71 ± 0.46	11.73 ± 1.11
	180	105.60 ± 0.30	13.84 ± 0.84
240	30	105.42 ± 1.62	11.00 ± 3.14
	90	107.68 ± 1.03	16.01 ± 1.27
	180	110.50 ± 2.78	22.87 ± 6.55
300	30	106.75 ± 0.43	8.86 ± 0.58
	90	113.80 ± 0.31	17.59 ± 0.37
	180	114.66 ± 0.22	18.81 ± 0.12

## Data Availability

All data generated and used in the study are available in the article and Supplementary File.

## Supplementary Materials

The following supporting information can be downloaded at: https://www.mdpi.com/article/10.3390/ma16216903/s1.

## Abbreviations

*AC*	Ash content
*C*	Carbon content
*EDr*	Energy densification ratio
*EG*	Energy gain
*Eu*	Energy usage
*Eue*	Energy usage to one unit of energy available in One unit of obtained hydrochar
*EY*	Energy yield
*FC*	Fixed carbon
*FR*	Fuel ratio
*H*	Hydrogen content
*H/C*	Hydrogen/carbon ratio
*HC*	Hydrochar
*HHV*	High heating value
*HTC*	Hydrothermal carbonization
*LHV*	Low heating value
*MC*	Moisture content
*MY*	Mass yield
*N*	Nitrogen content
*O*	Oxygen content
*O/C*	Oxygen/carbon ratio
*S*	Sulfur content
*SS*	Sewage sludge
*VM*	Volatile matter

## Appendix A

Appendix A contains a statistical analysis of the results presented in the article. The bold font signifies a statistically significant difference (*p* < 0.05) between particular process parameters, e.g., 180–30 refers to the process conducted at 180 °C in 30 min.

**Table A1:** Two-way analysis of variance (ANOVA) with post hoc Tukey test for VM content; the bold font signifies a statistically significant difference (*p* < 0.05).

Temp–Time	180–30	180–90	180–180	240–30	240–90	240–180	300–30	300–90	300–180
180–30		0.980421	**0.008373**	**0.000173**	**0.000173**	**0.000173**	**0.000173**	**0.000173**	**0.000173**
180–90	0.980421		0.062978	**0.000173**	**0.000173**	**0.000173**	**0.000173**	**0.000173**	**0.000173**
180–180	**0.008373**	0.062978		**0.000173**	**0.000173**	**0.000175**	**0.000173**	**0.000173**	**0.000173**
240–30	**0.000173**	**0.000173**	**0.000173**		0.282171	**0.005293**	**0.000173**	**0.000173**	**0.000173**
240–90	**0.000173**	**0.000173**	**0.000173**	0.282171		0.508906	**0.000173**	**0.000173**	**0.000173**
240–180	**0.000173**	**0.000173**	**0.000175**	**0.005293**	0.508906		**0.000173**	**0.000173**	**0.000173**
300–30	**0.000173**	**0.000173**	**0.000173**	**0.000173**	**0.000173**	**0.000173**		0.710134	0.876884
300–90	**0.000173**	**0.000173**	**0.000173**	**0.000173**	**0.000173**	**0.000173**	0.710134		0.094689
300–180	**0.000173**	**0.000173**	**0.000173**	**0.000173**	**0.000173**	**0.000173**	0.876884	0.094689	

**Table A2:** Two-way analysis of variance (ANOVA) with post hoc Tukey test for FC content; the bold font signifies a statistically significant difference (*p* < 0.05).

Temp–Time	180–30	180–90	180–180	240–30	240–90	240–180	300–30	300–90	300–180
180–30		0.700510	0.659952	0.576140	0.976754	0.967199	0.050718	**0.049810**	**0.000514**
180–90	0.700510		1.000000	**0.031117**	0.997476	0.998663	**0.001471**	**0.001446**	**0.000180**
180–180	0.659952	1.000000		**0.027111**	0.995342	0.997341	**0.001295**	**0.001274**	**0.000179**
240–30	0.576140	**0.031117**	**0.027111**		0.126318	0.112739	0.832027	0.827661	**0.021851**
240–90	0.976754	0.997476	0.995342	0.126318		1.000000	**0.006277**	**0.006160**	**0.000219**
240–180	0.967199	0.998663	0.997341	0.112739	1.000000		**0.005521**	**0.005419**	**0.000215**
300–30	0.050718	**0.001471**	**0.001295**	0.832027	**0.006277**	**0.005521**		1.000000	0.340606
300–90	**0.049810**	**0.001446**	**0.001274**	0.827661	**0.006160**	**0.005419**	1.000000		0.345111
300–180	**0.000514**	**0.000180**	**0.000179**	**0.021851**	**0.000219**	**0.000215**	0.340606	0.345111	

**Table A3:** Two-way analysis of variance (ANOVA) with post hoc Tukey test for FR; the bold font signifies a statistically significant difference (*p* < 0.05).

Temp–Time	180–30	180–90	180–180	240–30	240–90	240–180	300–30	300–90	300–180
180–30		0.793802	0.797532	0.297801	0.999633	0.998355	**0.004931**	**0.006017**	**0.000195**
180–90	0.793802		1.000000	**0.015479**	0.976154	0.989466	**0.000337**	**0.000373**	**0.000174**
180–180	0.797532	1.000000		**0.015711**	0.977135	0.989990	**0.000339**	**0.000376**	**0.000174**
240–30	0.297801	**0.015479**	**0.015711**		0.117537	0.092714	0.467936	0.525054	**0.003757**
240–90	0.999633	0.976154	0.977135	0.117537		1.000000	**0.001631**	**0.001976**	**0.000178**
240–180	0.998355	0.989466	0.989990	0.092714	1.000000		**0.001281**	**0.001536**	**0.000176**
300–30	**0.004931**	**0.000337**	**0.000339**	0.467936	**0.001631**	**0.001281**		1.000000	0.241881
300–90	**0.006017**	**0.000373**	**0.000376**	0.525054	**0.001976**	**0.001536**	1.000000		0.206552
300–180	**0.000195**	**0.000174**	**0.000174**	**0.003757**	**0.000178**	**0.000176**	0.241881	0.206552	

**Table A4:** Two-way analysis of variance (ANOVA) with post hoc Tukey test for AC; the bold font signifies a statistically significant difference (*p* < 0.05).

Temp–Time	180–30	180–90	180–180	240–30	240–90	240–180	300–30	300–90	300–180
180–30		0.188748	**0.000439**	**0.000173**	**0.000173**	**0.000173**	**0.000173**	**0.000173**	**0.000173**
180–90	0.188748		0.083802	**0.000173**	**0.000173**	**0.000173**	**0.000173**	**0.000173**	**0.000173**
180–180	**0.000439**	0.083802		**0.000177**	**0.000174**	**0.000227**	**0.000173**	**0.000173**	**0.000173**
240–30	**0.000173**	**0.000173**	**0.000177**		0.996438	0.964204	**0.000173**	**0.000176**	**0.000174**
240–90	**0.000173**	**0.000173**	**0.000174**	0.996438		0.630522	**0.000174**	**0.000195**	**0.000178**
240–180	**0.000173**	**0.000173**	**0.000227**	0.964204	0.630522		**0.000173**	**0.000174**	**0.000173**
300–30	**0.000173**	**0.000173**	**0.000173**	**0.000173**	**0.000174**	**0.000173**		0.768890	0.952915
300–90	**0.000173**	**0.000173**	**0.000173**	**0.000176**	**0.000195**	**0.000174**	0.768890		0.999891
300–180	**0.000173**	**0.000173**	**0.000173**	**0.000174**	**0.000178**	**0.000173**	0.952915	0.999891	

**Table A5:** Two-way analysis of variance (ANOVA) with post hoc Tukey test for C content; the bold font signifies a statistically significant difference (*p* < 0.05).

Temp–Time	180–30	180–90	180–180	240–30	240–90	240–180	300–30	300–90	300–180
180–30		0.274562	0.103497	0.768225	0.124147	**0.000285**	0.490891	**0.000173**	**0.000233**
180–90	0.274562		**0.000727**	**0.012554**	**0.000863**	**0.000174**	0.999953	**0.000173**	**0.000173**
180–180	0.103497	**0.000727**		0.853601	1.000000	0.071586	**0.001541**	**0.000239**	**0.037804**
240–30	0.768225	**0.012554**	0.853601		0.894030	**0.003713**	**0.029727**	**0.000175**	**0.001947**
240–90	0.124147	**0.000863**	1.000000	0.894030		0.059044	**0.001874**	**0.000228**	**0.030946**
240–180	**0.000285**	**0.000174**	0.071586	**0.003713**	0.059044		**0.000174**	0.060978	0.999994
300–30	0.490891	0.999953	**0.001541**	**0.029727**	**0.001874**	**0.000174**		**0.000173**	**0.000174**
300–90	**0.000173**	**0.000173**	**0.000239**	**0.000175**	**0.000228**	0.060978	**0.000173**		0.112724
300–180	**0.000233**	**0.000173**	**0.037804**	**0.001947**	**0.030946**	0.999994	**0.000174**	0.112724	

**Table A6:** Two-way analysis of variance (ANOVA) with post hoc Tukey test for H content; the bold font signifies a statistically significant difference (*p* < 0.05).

Temp–Time	180–30	180–90	180–180	240–30	240–90	240–180	300–30	300–90	300–180
180–30		0.777953	0.844167	0.714633	0.828613	0.999984	0.288964	0.995644	0.995644
180–90	0.777953		0.102935	1.000000	1.000000	0.562073	0.991330	0.993935	0.993935
180–180	0.844167	0.102935		0.083263	0.123605	0.962455	**0.018367**	0.396392	0.396392
240–30	0.714633	1.000000	0.083263		1.000000	0.494187	0.996553	0.986214	0.986214
240–90	0.828613	1.000000	0.123605	1.000000		0.622990	0.982917	0.997470	0.997470
240–180	0.999984	0.562073	0.962455	0.494187	0.622990		0.158505	0.954353	0.954353
300–30	0.288964	0.991330	**0.018367**	0.996553	0.982917	0.158505		0.729988	0.729988
300–90	0.995644	0.993935	0.396392	0.986214	0.997470	0.954353	0.729988		1.000000
300–180	0.995644	0.993935	0.396392	0.986214	0.997470	0.954353	0.729988	1.000000	

**Table A7:** Two-way analysis of variance (ANOVA) with post hoc Tukey test for N content; the bold font signifies a statistically significant difference (*p* < 0.05).

Temp–Time	180–30	180–90	180–180	240–30	240–90	240–180	300–30	300–90	300–180
180–30		**0.016942**	0.056144	**0.036678**	**0.000608**	**0.000179**	**0.000195**	**0.000199**	**0.000174**
180–90	**0.016942**		0.999388	0.999978	0.719618	**0.037289**	0.083552	0.103725	**0.001851**
180–180	0.056144	0.999388		1.000000	0.372191	**0.011069**	**0.025838**	**0.032655**	**0.000640**
240–30	**0.036678**	0.999978	1.000000		0.491439	**0.017232**	**0.039834**	0.050109	**0.000915**
240–90	**0.000608**	0.719618	0.372191	0.491439		0.610946	0.841063	0.889676	0.059878
240–180	**0.000179**	**0.037289**	**0.011069**	**0.017232**	0.610946		0.999959	0.999741	0.844842
300–30	**0.000195**	0.083552	**0.025838**	**0.039834**	0.841063	0.999959		1.000000	0.615979
300–90	**0.000199**	0.103725	**0.032655**	0.050109	0.889676	0.999741	1.000000		0.545625
300–180	**0.000174**	**0.001851**	**0.000640**	**0.000915**	0.059878	0.844842	0.615979	0.545625	

**Table A8:** Two-way analysis of variance (ANOVA) with post hoc Tukey test for S content; the bold font signifies a statistically significant difference (*p* < 0.05).

Temp–Time	180–30	180–90	180–180	240–30	240–90	240–180	300–30	300–90	300–180
180–30		1.000000	0.998589	0.962148	0.999455	0.995347	0.999965	1.000000	0.798099
180–90	1.000000		0.991474	0.988515	0.999977	0.980294	0.999298	0.999970	0.889051
180–180	0.998589	0.991474		0.680948	0.928772	1.000000	0.999999	0.999885	0.408673
240–30	0.962148	0.988515	0.680948		0.999737	0.605101	0.825838	0.911404	0.999908
240–90	0.999455	0.999977	0.928772	0.999737		0.886383	0.981119	0.995868	0.981119
240–180	0.995347	0.980294	1.000000	0.605101	0.886383		0.999974	0.999354	0.343721
300–30	0.999965	0.999298	0.999999	0.825838	0.981119	0.999974		1.000000	0.562652
300–90	1.000000	0.999970	0.999885	0.911404	0.995868	0.999354	1.000000		0.689222
300–180	0.798099	0.889051	0.408673	0.999908	0.981119	0.343721	0.562652	0.689222	

**Table A9:** Two-way analysis of variance (ANOVA) with post hoc Tukey test for O content; the bold font signifies a statistically significant difference (*p* < 0.05).

Temp–Time	180–30	180–90	180–180	240–30	240–90	240–180	300–30	300–90	300–180
180–30		0.713264	**0.000339**	**0.000173**	**0.000173**	**0.000173**	**0.000173**	**0.000173**	**0.000173**
180–90	0.713264		**0.000176**	**0.000173**	**0.000173**	**0.000173**	**0.000173**	**0.000173**	**0.000173**
180–180	**0.000339**	**0.000176**		**0.012927**	**0.002203**	**0.000485**	**0.000269**	**0.000173**	**0.000173**
240–30	**0.000173**	**0.000173**	**0.012927**		0.991600	0.704855	0.370026	**0.000173**	**0.000173**
240–90	**0.000173**	**0.000173**	**0.002203**	0.991600		0.991260	0.858435	**0.000173**	**0.000174**
240–180	**0.000173**	**0.000173**	**0.000485**	0.704855	0.991260		0.999529	**0.000173**	**0.000182**
300–30	**0.000173**	**0.000173**	**0.000269**	0.370026	0.858435	0.999529		**0.000174**	**0.000219**
300–90	**0.000173**	**0.000173**	**0.000173**	**0.000173**	**0.000173**	**0.000173**	**0.000174**		0.542097
300–180	**0.000173**	**0.000173**	**0.000173**	**0.000173**	**0.000174**	**0.000182**	**0.000219**	0.542097	

**Table A10:** Two-way analysis of variance (ANOVA) with post hoc Tukey test for HHV; the bold font signifies a statistically significant difference (*p* < 0.05).

Temp–Time	180–30	180–90	180–180	240–30	240–90	240–180	300–30	300–90	300–180
180–30		1.000000	0.994505	0.998802	0.248931	**0.002294**	0.678189	**0.000177**	**0.000174**
180–90	1.000000		0.996346	0.999303	0.268364	**0.002521**	0.706494	**0.000178**	**0.000174**
180–180	0.994505	0.996346		1.000000	0.690270	**0.012058**	0.982840	**0.000211**	**0.000178**
240–30	0.998802	0.999303	1.000000		0.593045	**0.008649**	0.958209	**0.000197**	**0.000177**
240–90	0.248931	0.268364	0.690270	0.593045		0.327477	0.995357	**0.001470**	**0.000443**
240–180	**0.002294**	**0.002521**	**0.012058**	**0.008649**	0.327477		0.084799	0.170501	**0.043083**
300–30	0.678189	0.706494	0.982840	0.958209	0.995357	0.084799		**0.000411**	**0.000229**
300–90	**0.000177**	**0.000178**	**0.000211**	**0.000197**	**0.001470**	0.170501	**0.000411**		0.997282
300–180	**0.000174**	**0.000174**	**0.000178**	**0.000177**	**0.000443**	**0.043083**	**0.000229**	0.997282	

**Table A11:** Two-way analysis of variance (ANOVA) with post hoc Tukey test for LHV; the bold font signifies a statistically significant difference (*p* < 0.05).

Temp–Time	180–30	180–90	180–180	240–30	240–90	240–180	300–30	300–90	300–180
180–30		0.999997	0.999388	0.988581	0.170389	**0.002523**	0.454337	**0.000176**	**0.000174**
180–90	0.999997		0.999996	0.999143	0.273255	**0.004513**	0.627142	**0.000179**	**0.000174**
180–180	0.999388	0.999996		0.999995	0.423332	**0.008463**	0.802077	**0.000191**	**0.000175**
240–30	0.988581	0.999143	0.999995		0.610938	**0.016289**	0.930075	**0.000209**	**0.000178**
240–90	0.170389	0.273255	0.423332	0.610938		0.475432	0.998848	**0.001788**	**0.000507**
240–180	**0.002523**	**0.004513**	**0.008463**	**0.016289**	0.475432		0.181332	0.125002	**0.030227**
300–30	0.454337	0.627142	0.802077	0.930075	0.998848	0.181332		**0.000574**	**0.000261**
300–90	**0.000176**	**0.000179**	**0.000191**	**0.000209**	**0.001788**	0.125002	**0.000574**		0.997258
300–180	**0.000174**	**0.000174**	**0.000175**	**0.000178**	**0.000507**	**0.030227**	**0.000261**	0.997258	

**Table A12:** Two-way analysis of variance (ANOVA) with post hoc Tukey test for EY; the bold font signifies a statistically significant difference (*p* < 0.05).

Temp–Time	180–30	180–90	180–180	240–30	240–90	240–180	300–30	300–90	300–180
180–30		0.265994	0.305547	**0.000174**	**0.000233**	**0.004340**	**0.000173**	**0.000173**	**0.000173**
180–90	0.265994		1.000000	**0.000562**	**0.012248**	0.474336	**0.000173**	**0.000173**	**0.000173**
180–180	0.305547	1.000000		**0.000494**	**0.010160**	0.422832	**0.000173**	**0.000173**	**0.000173**
240–30	**0.000174**	**0.000562**	**0.000494**		0.778252	**0.035037**	**0.000173**	**0.000173**	**0.000173**
240–90	**0.000233**	**0.012248**	**0.010160**	0.778252		0.528171	**0.000173**	**0.000173**	**0.000173**
240–180	**0.004340**	0.474336	0.422832	**0.035037**	0.528171		**0.000173**	**0.000173**	**0.000173**
300–30	**0.000173**	**0.000173**	**0.000173**	**0.000173**	**0.000173**	**0.000173**		0.999255	1.000000
300–90	**0.000173**	**0.000173**	**0.000173**	**0.000173**	**0.000173**	**0.000173**	0.999255		0.999828
300–180	**0.000173**	**0.000173**	**0.000173**	**0.000173**	**0.000173**	**0.000173**	1.000000	0.999828	

**Table A13:** Two-way analysis of variance (ANOVA) with post hoc Tukey test for MY; the bold font signifies a statistically significant difference (*p* < 0.05).

Temp–Time	180–30	180–90	180–180	240–30	240–90	240–180	300–30	300–90	300–180
180–30		0.156140	0.092846	**0.000173**	**0.000174**	**0.000181**	**0.000173**	**0.000173**	**0.000173**
180–90	0.156140		0.999998	**0.000233**	**0.000580**	**0.004472**	**0.000173**	**0.000173**	**0.000173**
180–180	0.092846	0.999998		**0.000276**	**0.000927**	**0.008056**	**0.000173**	**0.000173**	**0.000173**
240–30	**0.000173**	**0.000233**	**0.000276**		0.985301	0.518420	**0.000173**	**0.000173**	**0.000173**
240–90	**0.000174**	**0.000580**	**0.000927**	0.985301		0.964426	**0.000173**	**0.000173**	**0.000173**
240–180	**0.000181**	**0.004472**	**0.008056**	0.518420	0.964426		**0.000173**	**0.000173**	**0.000173**
300–30	**0.000173**	**0.000173**	**0.000173**	**0.000173**	**0.000173**	**0.000173**		0.703077	0.894706
300–90	**0.000173**	**0.000173**	**0.000173**	**0.000173**	**0.000173**	**0.000173**	0.703077		0.999974
300–180	**0.000173**	**0.000173**	**0.000173**	**0.000173**	**0.000173**	**0.000173**	0.894706	0.999974	

**Table A14:** Two-way analysis of variance (ANOVA) with post hoc Tukey test for EDr; the bold font signifies a statistically significant difference (*p* < 0.05).

Temp–Time	180–30	180–90	180–180	240–30	240–90	240–180	300–30	300–90	300–180
180–30		1.000000	0.994505	0.998802	0.248931	**0.002294**	0.678189	**0.000177**	**0.000174**
180–90	1.000000		0.996346	0.999303	0.268364	**0.002521**	0.706494	**0.000178**	**0.000174**
180–180	0.994505	0.996346		1.000000	0.690270	**0.012058**	0.982840	**0.000211**	**0.000178**
240–30	0.998802	0.999303	1.000000		0.593045	**0.008649**	0.958209	**0.000197**	**0.000177**
240–90	0.248931	0.268364	0.690270	0.593045		0.327477	0.995357	**0.001470**	**0.000443**
240–180	**0.002294**	**0.002521**	**0.012058**	**0.008649**	0.327477		0.084799	0.170501	**0.043083**
300–30	0.678189	0.706494	0.982840	0.958209	0.995357	0.084799		**0.000411**	**0.000229**
300–90	**0.000177**	**0.000178**	**0.000211**	**0.000197**	**0.001470**	0.170501	**0.000411**		0.997282
300–180	**0.000174**	**0.000174**	**0.000178**	**0.000177**	**0.000443**	**0.043083**	**0.000229**	0.997282	

**Table A15:** Two-way analysis of variance (ANOVA) with post hoc Tukey test for EG; the bold font signifies a statistically significant difference (*p* < 0.05).

Temp–Time	180–30	180–90	180–180	240–30	240–90	240–180	300–30	300–90	300–180
180–30		0.999958	0.999962	0.998084	0.927790	**0.019611**	0.818977	0.628027	0.354202
180–90	0.999958		0.994088	0.999998	0.745434	**0.008339**	0.961519	0.385747	0.185138
180–180	0.999962	0.994088		0.963760	0.992764	**0.044761**	0.585379	0.851691	0.586582
240–30	0.998084	0.999998	0.963760		0.576901	**0.004663**	0.993507	0.254132	0.112290
240–90	0.927790	0.745434	0.992764	0.576901		0.213789	0.176591	0.999231	0.966605
240–180	**0.019611**	**0.008339**	**0.044761**	**0.004663**	0.213789		**0.000929**	0.512172	0.792095
300–30	0.818977	0.961519	0.585379	0.993507	0.176591	**0.000929**		0.056843	**0.021867**
300–90	0.628027	0.385747	0.851691	0.254132	0.999231	0.512172	0.056843		0.999880
300–180	0.354202	0.185138	0.586582	0.112290	0.966605	0.792095	**0.021867**	0.999880	

**Table A16:** Two-way analysis of variance (ANOVA) with post hoc Tukey test for Eu; the bold font signifies a statistically significant difference (*p* < 0.05).

Temp–Time	180–30	180–90	180–180	240–30	240–90	240–180	300–30	300–90	300–180
180–30		0.997924	0.320661	**0.034421**	**0.009685**	**0.001406**	**0.000173**	**0.000173**	**0.000173**
180–90	0.997924		0.723579	0.133218	**0.040924**	**0.005732**	**0.000173**	**0.000173**	**0.000173**
180–180	0.320661	0.723579		0.932714	0.635075	0.167843	**0.000173**	**0.000173**	**0.000173**
240–30	**0.034421**	0.133218	0.932714		0.999179	0.795439	**0.000173**	**0.000173**	**0.000173**
240–90	**0.009685**	**0.040924**	0.635075	0.999179		0.984252	**0.000173**	**0.000173**	**0.000173**
240–180	**0.001406**	**0.005732**	0.167843	0.795439	0.984252		**0.000173**	**0.000173**	**0.000173**
300–30	**0.000173**	**0.000173**	**0.000173**	**0.000173**	**0.000173**	**0.000173**		**0.000268**	**0.000173**
300–90	**0.000173**	**0.000173**	**0.000173**	**0.000173**	**0.000173**	**0.000173**	**0.000268**		**0.002710**
300–180	**0.000173**	**0.000173**	**0.000173**	**0.000173**	**0.000173**	**0.000173**	**0.000173**	**0.002710**	

**Table A17:** Two-way analysis of variance (ANOVA) with post hoc Tukey test for Eue; the bold font signifies a statistically significant difference (*p* < 0.05).

Temp–Time	180–30	180–90	180–180	240–30	240–90	240–180	300–30	300–90	300–180
180–30		0.996201	0.247857	**0.019062**	**0.007034**	**0.001465**	**0.000173**	**0.000173**	**0.000173**
180–90	0.996201		0.662700	0.087972	**0.034194**	**0.006849**	**0.000173**	**0.000173**	**0.000173**
180–180	0.247857	0.662700		0.892702	0.643274	0.230586	**0.000173**	**0.000173**	**0.000173**
240–30	**0.019062**	0.087972	0.892702		0.999866	0.924886	**0.000173**	**0.000173**	**0.000173**
240–90	**0.007034**	**0.034194**	0.643274	0.999866		0.995743	**0.000173**	**0.000173**	**0.000173**
240–180	**0.001465**	**0.006849**	0.230586	0.924886	0.995743		**0.000173**	**0.000173**	**0.000173**
300–30	**0.000173**	**0.000173**	**0.000173**	**0.000173**	**0.000173**	**0.000173**		**0.002689**	**0.000173**
300–90	**0.000173**	**0.000173**	**0.000173**	**0.000173**	**0.000173**	**0.000173**	**0.002689**		**0.004338**
300–180	**0.000173**	**0.000173**	**0.000173**	**0.000173**	**0.000173**	**0.000173**	**0.000173**	**0.004338**	

## Appendix B

Appendix B contains graphs presenting temperature and pressure patterns during the HTC process with different parameters.

Figure A1, Figure A2 and Figure A3 present the temperature and pressure patterns for the HTC process performed at 180 °C for 30, 90, and 180 min, respectively.

Figure A4, Figure A5 and Figure A6 present the temperature and pressure patterns for the HTC process performed at 240 °C for 30, 90, and 180 min, respectively.

Figure A7 and Figure A8 present the temperature and pressure patterns for the HTC process performed at 300 °C for 30, 90, min, respectively.
